# Systemic inflammatory responses to repeated and increasing endotoxin challenges in fetal sheep

**DOI:** 10.14814/phy2.70316

**Published:** 2025-04-23

**Authors:** Sanya Kathuria, Akash Gupta, Ayna R. Tracy, Rosa I. Luna Ramirez, Senthil Kumar Thulasingam, Nahla Zaghloul, Mohamed Ahmed, Sean W. Limesand

**Affiliations:** ^1^ School of Animal and Comparative Biomedical Sciences University of Arizona Tucson Arizona USA; ^2^ Division of Neonatology, Department of Pediatrics, College of Medicine University of Arizona Tucson Arizona USA; ^3^ Division of Neonatology, Department of Pediatrics, College of Medicine University of Florida Gainesville Florida USA

**Keywords:** chorioamnionitis, FIRS, lipopolysaccharide, transcriptomic sequencing

## Abstract

Repeated low‐dose administration of lipopolysaccharide (LPS) attenuates subsequent fetal responses, which makes it challenging to investigate interventions to prolonged exposure. Our aim was to develop a fetal inflammatory response syndrome (FIRS) model that consistently and effectively elicits a marked physiological response to increasing LPS doses. Four intravenous LPS boluses (0.3, 1.5, 3, and 15 μg) were administered to fetal sheep over 5 days. Physiological responses were measured via blood gases, pH, lactate, and cortisol concentrations. Fetal peripheral blood mononuclear cells (PBMCs) were analyzed for transcriptomic changes and tissue cytokine expression postmortem. All LPS challenges increased lactate, cortisol, and pCO_2_ concentrations and decreased pH and pO_2_ levels at 3 and 5 hours. No interaction was found between day (increasing LPS doses) and hour (LPS response to each dose). PBMC numbers increase with LPS challenges. Transcriptional analysis on PBMCs identified several enriched gene clusters indicating upregulation of inflammatory gene signatures along with complement activation and NFκB signaling pathways. Expression of pro‐inflammatory cytokines (TNFα, IL‐6, or IL‐1β) was measured in lung, heart, liver, placenta, and spleen. Physiological indices show both respiratory and metabolic acidosis with successive and increasing LPS challenges that demonstrate a robust systemic response despite tachyphylaxis to LPS in fetal sheep.

## Graphical abstract

Establishment of an ovine fetal inflammatory response syndrome model. We developed a novel ovine model of fetal inflammatory response syndrome (FIRS) using repeated low‐dose administration of lipopolysaccharide (LPS). Our approach reliably induces a robust physiological response, including respiratory and metabolic acidosis, despite the presence of tachyphylaxis with increasing LPS exposure. Transcriptomic analysis of fetal peripheral blood mononuclear cells revealed enriched gene clusters associated with inflammation, complement activation, and NFκB signaling pathways ‐ key features of systemic fetal inflammation. Activation of these inflammatory cascades plays a central role in driving adverse outcomes in fetuses exposed to chorioamnionitis. The ovine model replicates several aspects of human FIRS and provides a consistent and controlled platform for investigating fetal immune responses to chorioamnionitis.

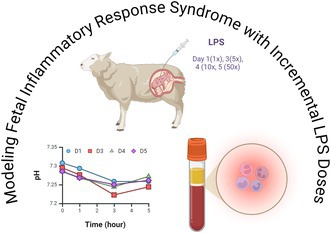

## 1 INTRODUCTION

Chorioamnionitis, observed in 3%–5% of pregnancies, is a leading cause of preterm births before 24 weeks of gestation and is characterized by inflammation of intrauterine structures such as the placenta, chorion, and amnion (Ericson & Laughon, 2015; Jain et al., 2022; Kachikis et al., 2019; Ramsey et al., 2005). This condition causes a systemic inflammatory response in the fetus, marked by elevated pro‐inflammatory cytokines like interleukin 6 (IL‐6), tumor necrosis factor alpha (TNFα), IL‐1β and white blood cell counts that result in fetal inflammatory response syndrome (FIRS) (Cappelletti et al., 2020; Gomez et al., 1994; Hillier et al., 1993; Jung et al., 2020). FIRS is also postulated to contribute to neonatal morbidities like bronchopulmonary dysplasia, necrotizing enterocolitis, and intracerebral hemorrhage (Ahmed et al., 2023; Muraskas et al., 2020). Despite current antibiotic interventions, inadequate coverage against pathogens leaves opportunities for persistent infection and inflammation of the fetus (Chapman et al., 2014; Lukanovic et al., 2023).

Numerous animal models of chorioamnionitis have been established to investigate the disease pathogenesis, consequences of FIRS, and potential clinical interventions (Ahmed et al., 2023; Brosius Lutz et al., 2021). Experiments in fetal sheep have provided valuable information on the fetal inflammatory response and its short‐ and long‐term implications for organ development and function (Hillman et al., 2024; Hoogenboom et al., 2022; Inocencio et al., 2022; Zarate et al., 2020). Lipopolysaccharides (LPS) were administered either intra‐amniotically or intravenously to elicit a robust systemic inflammatory response in fetal sheep that affects their physiology and organ development (Duncan et al., 2002; Jobe et al., 2000; Mathai et al., 2013; Zarate et al., 2020). Although both routes of LPS administration induce FIRS, reports in the literature reveal a wider range of doses for intra‐amniotic administration (1–10 mg/kg) compared to intravenous administration (0.1–1.5 μg/kg), which reflects the direct fetal administration of LPS versus the dilution in the amniotic fluid despite being more relevant for intra‐amniotic infections (Disdier et al., 2020; Lear et al., 2014; Moss et al., 2002; Zarate et al., 2020). Irrespective of the administration site, repeated subacute doses, reminiscent of slowly progressing infection, result in an attenuated response to subsequent LPS doses (Disdier et al., 2020; Duncan et al., 2002; Lear et al., 2014; Mathai et al., 2013).

Tachyphylaxis with successive LPS challenges lowers the dynamic range of the subsequent physiological responses and makes it difficult to assess whether clinical interventions to prolong established intrauterine infections improve fetal outcomes with treatment to immunomodulatory drugs (Ahmed et al., 2023; Mathai et al., 2013). Thus, there is a need to establish a new model that replicates this clinical scenario and can be used to study immunomodulatory drug response over a longer period of exposure. In this study, our goal was to develop a five‐day FIRS model that consistently and effectively elicits a robust physiological response to increasing LPS doses in pre‐term sheep fetuses. We hypothesize that repeated increasing doses of LPS when given intravenously to the fetus will overcome tachyphylaxis induced by repeated LPS exposure and cause a systemic inflammatory response in fetal sheep. Progressively increasing LPS boluses were administered because earlier reports showed initial low‐dose boluses decreased mortality in preterm sheep (Lear et al., 2014; Mathai et al., 2013). The duration of 5 days was based on clinical observations indicating that mothers experiencing chorioamnionitis should have an expedited delivery (Conde‐Agudelo et al., 2020). Therefore, our new approach replicates the clinical setting of a pregnant woman presenting with chorioamnionitis, which is associated with prolonged fetal inflammation and preterm delivery in humans (Brosius Lutz et al., 2021; Romero et al., 2006).

## 2 MATERIALS AND METHODS

### 2.1 Surgical preparation

Columbia‐Rambouillet crossbred ewes pregnant with singletons were managed according to the guidelines set by the Guide for The Care and Use of Laboratory Animals, National Research Council, and approved by the Institutional Animal Care and Use Committee (Protocol #08–132). Pregnant ewes (*n* = 5) were shipped to the Agricultural Research Center and kept at 22 ± 2°C for 5 days prior to surgery. Ewes were fed Standard Bread Alfalfa Pellets (Sacate Pellet Mills) and provided water and salt ad libitum. At 119 ± 1 days gestational age (dGA) the ewe and fetus underwent a surgical procedure to place indwelling fetal and maternal vascular catheters (Tygon ND‐100‐80 Flexible Plastic Tubing) as previously described by (Davis et al., 2020). A total of six catheters were placed: four in the fetus (two hind pedal arterial and two hind pedals veins) and two in the ewe (femoral arterial and vein). Vascular catheters were flushed daily with heparinized saline solution (100 U/mL heparin in 0.9% NaCl solution, Vedco, Inc., St. Joseph, MO).

### 2.2 Experimental design

**FIGURE 1:**
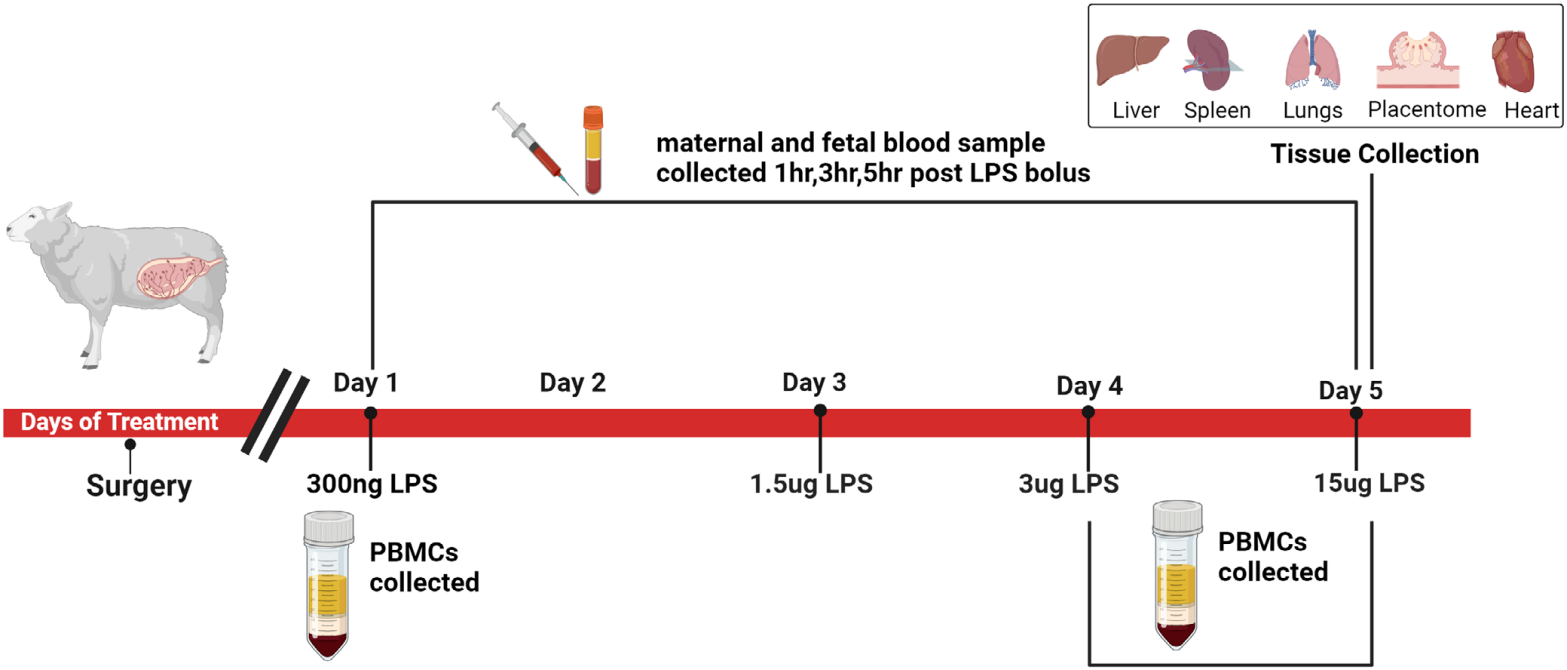
Experimental timeline for LPS challenges. Fetal and maternal catheters were surgically placed at least 3 days prior to treatment. LPS was administered on four separate days with increasing concentrations that are defined on the timeline (Days of Treatment). During each LPS challenge, maternal and fetal blood were collected before the LPS bolus (time 0) and at 1, 3, and 5 h after giving LPS. Peripheral blood mononuclear cells (PBMCs) were collected and isolated on Day 1, 4, and 5 at time 0 prior to that daily LPS bolus. After completing the final LPS challenge, fetal organs (lungs, heart, liver, spleen, and placentome [cotyledon]) were measured for cytokine mRNA expression. Created in https://BioRender.com.

Beginning at 123 ± 2 dGA, intravenous (IV) LPS (E. coli serotype O55:B5) boluses were administered to the fetus. Four increasing LPS boluses dissolved in saline were given (0.3 ug, 1.5 ug, 3 ug,15 ug) on Day 1, Day 3, Day 4, and Day 5, respectively (Figure 1). An LPS challenge on Day 2 was omitted to allow for the future administration of new interventions. Peripheral blood mononuclear cells (PBMCs) were collected and isolated on dGA (study day) 123 (Jain et al., 2022), 126 (Ramsey et al., 2005), and 127 (Jung et al., 2020) prior to the daily LPS bolus. During each LPS challenge, arterial blood pH and gases and plasma lactate concentrations were measured at hour 0 (pre‐bolus) and at hours 1, 3, and 5 (post‐LPS bolus). Plasma was also stored at −80°C for cortisol measurements. After the final LPS bolus (15 ug; Day 5), the ewe and fetus were humanely killed after the 5‐hour sample collection with an overdose of pentobarbital sodium (86 mg/kg, Euthasol; Virbac Animal Health, Fort Worth, TX). The fetus as well as fetal organs (lungs, heart, liver, spleen, and type b placentomes) were dissected, weighed, and snap frozen. RNA was isolated from PBMCs and tissues (lung, heart, liver, spleen, placenta [cotyledon]) for RNA sequencing and quantitative real‐time PCR (qPCR) analysis, respectively. Cytokine mRNA expression (IL‐6, TNF‐α, and IL1β) was measured with quantitative (q)PCR.

### 2.3 Blood sample collection and analysis

Arterial blood samples were collected for blood gas and pH and plasma lactate and hormone measurements. Blood samples collected in heparin‐lined 1 mL syringes were used to measure whole blood pH, gases, and oximetry with an ABL 825 Blood Gas Analyzer (Radiometer, Copenhagen, Denmark) as described by (Davis et al., 2021). Blood collected in 3 mL EDTA‐lined syringes was centrifuged (1000 × g) and plasma was stored for hormone measurements. Additionally, plasma lactate concentrations were measured with the YSI model 2900 SELECT Biochemistry Analyzer (YSI, Yellow Springs, OH). Cortisol concentrations were determined with a Cortisol EIA (Cat. # EA65, Oxford Biomedical Research, Rochester Hills, MI).

### 2.4 PBMC isolation

PBMCs were isolated from arterial blood using Ficoll‐Paque density gradient centrifugation. Whole blood (10 mL) collected in EDTA‐coated vacutainer tubes (BD Vacutainer® EDTA Tubes) was mixed and incubated at room temperature for 45 minutes. Blood was diluted 1:1 with PBS containing 2% FBS (Thermo Scientific™Dry‐blend Buffered Packs cat #28374) and layered over Ficoll‐Paque media (GE Healthcare cat #17–1440‐02), then centrifuged at 500 ***g*** for 30 min at 25°C. The PBMC layer was transferred, washed with serum‐free RPMI media (Gibco™ RPMI 1640 cat #3180–022), and centrifuged again at 500 ***g*** for 6 min. To reduce RBC contamination, RBC lysis buffer was added to the PBMC suspension and incubated for 10 min. After centrifugation at 500 ***g*** for 5 min, the pelleted cells were resuspended in RPMI + 40% FBS media and counted using a hemocytometer with Trypan blue staining (Invitrogen cat #T10282). The cell concentration was adjusted to 1 × 10^6^ cells/mL, aliquoted into centrifuge tubes, centrifuged, and the cell pellets were frozen at −80°C in preparation for RNA extraction.

### 2.5 RNA isolation and cDNA preparation

RNA was extracted from snap‐frozen tissues using the RNeasy Fibrous Mini Kit (Qiagen, Cat #74704) and concentrations were measured using Qubit (Invitrogen™ Qubit™ 4 Fluorometer). Integrity was determined at the University of Arizona Genetics Core Lab using Agilent 2100. The RNA samples with the RNA integrity >7 were used for qPCR. cDNA was prepared by reverse transcribing RNA (1ug) per reaction in duplicate with Superscript III reverse transcription (Thermo‐Fisher cat #18080044) using the instructions in the manual (Kelly et al., 2018). Fetal sheep exposed to LPS described in this study were compared to age‐matched control fetuses (*n* = 5) that were naïve to LPS treatments but received identical surgical procedures and daily heparin‐saline flushes for catheter maintenance.

### 2.6 Quantitative polymerized chain reaction (qPCR)

Synthetic oligonucleotides were designed with the aid of NCBI Primer Blast and purchased from Eurofins MWG Operon (Huntsville, AL) (Table 1). PCR products for ovine inflammatory markers (TNFα, IL‐6, and IL‐1β) were amplified from sheep fetal liver mRNA by reverse transcription‐PCR using Taq DNA Polymerase (Qiagen) according to the manufacturer's instructions. Optimal annealing temperature and product size were validated for each pair of primers. PCR products were cloned into a plasmid and confirmed with nucleotide sequencing as described previously (Kelly et al., 2018). Primer efficiencies and sensitivities were measured with serial cDNA dilutions, and only primers with efficiencies >85% and <100% were used.

**TABLE 1:** Quantitative PCR Primers.

Primer	Nucleotide sequence 5′ to 3′	Product size (bp)	Annealing temperature (C)
RPS15	F‐ATCATTCTGCCCGAGATGGTG R‐TGCTTTAGCGGGCTTGTAGGTG	134	60
GAPDH	F‐CTGGCCAAGGTCATCCAT R‐ ACAGTCTTCTGGGTGGCAGT	106	60
TBP	F‐AGAATAAGAGAGCCCCGCAC R‐ TTCACATCACAGCTCCCCAC	188	60
TNFα	F‐CTGGGCAGGTCTACTTTGGG R‐ GAAGGGGATGAGGAGGGTCT	109	60
IL‐6	F‐ACCTGGACTTCCTCCAGAAC R‐TTGAGCCGAGAAGTGGTGTTC	162	60
IL‐1β	F‐TGAGCCGAGAAGTGGTGTTC R‐ GCCACCTCTAAAACGTCCCA	314	62

Abbreviations: GAPDH, Glyceraldehyde 3‐phosphate dehydrogenase; IL‐1β, Interleukin‐1 beta; IL‐6, Interleukin 6; S15, Ribosomal protein S15; TBP, TATA‐binding protein; TNFα, Tumor Necrosis Factor‐alpha.

Relative expression of mRNA was determined with SYBR Green (Cat # 330500, Qiagen) in a CFX Connect Real‐Time PCR Detection System (Bio‐Rad, CFX Connect Real time System). The protocol began with initial denaturation done at 95°C for 15 min, followed by 40 cycles of 96°C for 30 s, annealing at 54–62°C (determined for each primer set) for 30 s, and 72°C for 10 s, with fluorescence measurement at the end of each cycle. Melt curve analysis was performed at the end of the amplification to confirm product homogeneity. Samples were run in duplicate; the results were normalized to the geometric mean of ovine reference genes: ribosomal protein S15 (S15), TATA‐binding protein (TBP) and/or Glyceraldehyde 3‐phosphate dehydrogenase (GAPDH). The results were analyzed by the comparative ΔCT method (CT gene of interest− CT geometric mean of reference genes). Average ΔCT was calculated, and fold change was calculated using 2^(‐ΔΔCT)^ (Livak & Schmittgen, 2001).

### 2.7 RNA sequencing for PBMCs

RNA from PBMCs was extracted with Tri‐Reagent (Cat # TR 118, Molecular Research Center, Cincinnati, OH) and cleaned up using a Qiagen Mini RNeasy column (Cat # 74104, Qiagen, Valencia, CA) (Luna‐Ramirez et al., 2023). RNA samples (RIN Score >7) isolated from fetal PBMCs (*n* = 5 fetuses) collected on Day 1, time 0 (prior to any LPS exposure, baseline) and Day 5, time 0 (after 3 LPS boluses) were submitted to the Novogene Corporation Inc. (Sacremento CA, USA) for high‐throughput transcriptome sequencing.

Raw sequence reads were subjected to quality control using FastQC software (version 0.11.9, accessed on March 14, 2024). Reads that passed the quality check were then aligned to reference genome of sheep (Ovis aries ARS‐UI_Ramb_v2.0 GenBank # GCA_016772045.1) downloaded from Ensembl Website (March 25, 2024) using The Spliced Transcripts Alignment to a Reference (STAR) software (version 2.7.3a). Since the RNA sequencing was strand specific, STAR was configured to consider strand information during alignment. FeatureCounts (v2.0.3) was used to quantify the reads mapped to the gene in specific strands. The count data from featureCounts was used for differential expression analysis using DESeq2 package (version1.44.0) accessed on R studio (version R.4.4.0). *p*‐values were adjusted using the Benjamini‐Hochberg method to control the false discovery rate. Differentially expressed genes (DEGs) were identified based on an adjusted *p*‐value threshold (*p*adj <0.05) and a log2 fold change criterion (absolute log2FC >1). The DEGs were input in Database for Annotation, Visualization and Integrated Discovery (DAVID; (Sherman et al., 2022)) accessed through National Institutes Health (NIH) to perform gene ontology (GO) enrichment for Biological Processes (GO: BP) and Molecular Function (GO:MF) and KEGG pathway analysis. Pathway enrichment found through DAVID was confirmed with gProfiler (Reimand et al., 2007). Pathways with adjusted *p*‐value <0.05 or FDR <0.05 were considered enriched or overrepresented.

### 2.8 Statistical analysis

A mixed two‐way ANOVA was performed to analyze the effects of day and hour on each physiological parameter (pH, pCO_2_(mmHg), pO_2_(mmHg), lactate (mmol/L), cortisol (ng/mL), PBMC (cells ×10^6^/mL)) and a Tukey–Kramer test was used to determine statistical significance differences (*p* < 0.05). A two‐tailed T‐Test was performed on ΔCT values between LPS naïve and LPS treated groups.

## 3 RESULTS

### 3.1 Increasing LPS doses elicited a repeated robust inflammatory response

**FIGURE 2:**
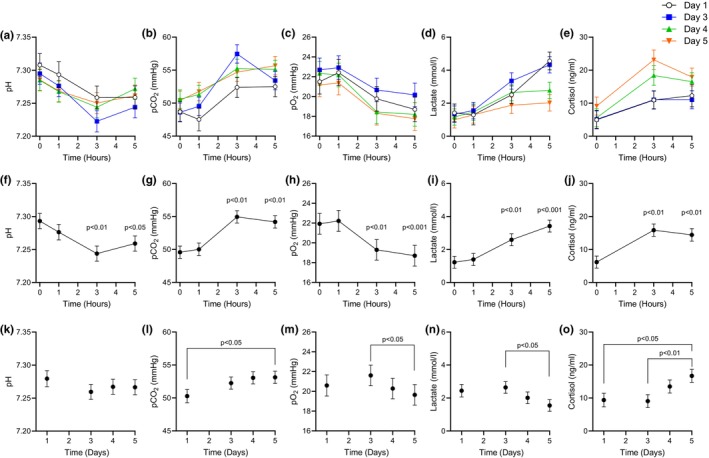
Physiological responses to repeated intravenous LPS challenges in fetuses (*n* = 5) for indicated hours and days. Blood pH, pCO_2_ (mmHg), pO_2_ (mmHg), and plasma lactate (mmol/L) and cortisol (ng/mL) are presented for individual challenges (panels a–e), averaged (±SEM) hour responses (panels f–j), and averaged (±SEM) day responses (k–o). Data were analyzed by two‐way mixed ANOVA for the main effects (hour and day) with sheep as the subject. Differences (*p* < 0.05) were determined with a Tukey–Kramer ‘s post hoc test, and significance levels are indicated.

Fetal physiological responses to the LPS challenge were assessed with pH, pCO_2_, pO_2_, lactate, and cortisol measurements at 1, 3, and 5 h post‐LPS bolus and compared to hour 0, which is the pre‐LPS bolus sample for each day (Figure 2a–e). Acute inflammatory responses were observed with each intravenous LPS challenge. However, there was no interaction between day (increasing LPS doses) and hour (LPS response to each dose) for any fetal parameter presented. Therefore, we present the main effects for hour means for all the LPS challenges and day means for each LPS challenge. Following the LPS bolus, the mean pH and pO_2_ decrease (*p* < 0.01) at 3‐ and 5‐h time points in comparison to hour 0 (Figure 2f,h). Conversely, the pCO_2_ levels, lactate concentrations, and cortisol concentrations increased (*p* < 0.01) at 3‐ and 5‐h time points compared to hour 0 (Figure 2g,i,j).

For each LPS challenge, the average daily response was calculated (i.e., day effect) as the average of all hourly measurements (Figure 2k–o). The blood pH was unaffected by the different daily LPS doses (Figure 2k). The daily pCO_2_ average was greater at Day 5 (15 μg) compared to Day 1 (0.3 μg) (Figure 2l). Daily pO_2_ and lactate averages decreased (*p* < 0.05) between Day 3 (1.5 μg) and Day 5 (15 μg) (Figure 2m,n). Daily cortisol concentration averages increased (*p* < 0.05) on Day 5 compared to Days 1 and 3 (Figure 2o).

The maternal blood pH, pCO_2_, pO_2_, and plasma lactate were measured during each fetal LPS challenge. In maternal samples, no differences were found between day, time, or their interaction (data not shown).

### 3.2 Activation of the innate immune response in fetal PBMCs

**FIGURE 3:**
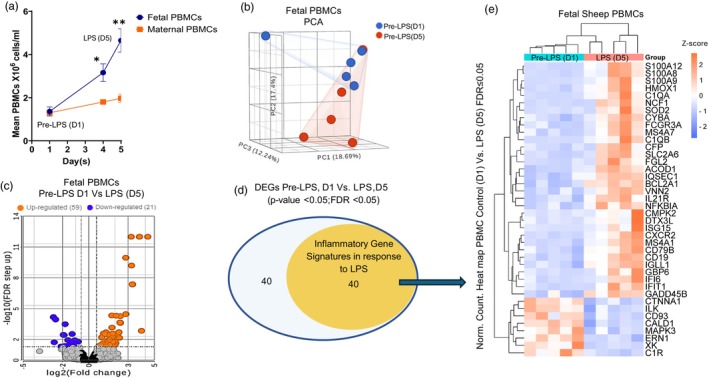
Fetal PBMC transcriptomic analysis reveals consistent upregulation of inflammatory gene signatures after LPS exposure. (a) Fetal PBMC concentrations (cells/mL) were determined prior to LPS exposure on Day 1 (Pre‐LPS(D1)) and on Day 4 and 5 (LPS D5). (b) Principal component analysis (PCA) for all transcriptome data for the pre‐LPS D1 (blue) and LPS D5 (red) are plotted based on their dimensional variance. (c) The volcano plot demonstrates DEGs (log2 Fold Change >1.5 and FDR *q* < 0.05) between the 2 days (D5 compared to D1). (d) The orange solid circle of the Venn Diagram indicates genes that were differentially expressed with LPS exposure and aligned with the inflammatory gene signature compared to the total numbers of DEGs encompassed in the purple circle. (e) Heat map illustrates the expression levels of the 40 DEGs associated with Inflammatory gene signatures in LPS response.

Repeated administration of LPS increased PBMC concentrations in fetal blood on Day 4 (*p* < 0.05) and Day 5 (*p* < 0.01) compared to the pre‐LPS bolus sample collected on Day 1 (Figure 3a). The fetal PBMCs increased ~2.4‐fold on Day 4 after the second LPS bolus (given on Day 3) and ~3.5 fold on Day 5 after the third LPS bolus (give on Day 4). Maternal PBMC concentrations were not different between Day 1 and Day 5 (Figure 3a).

The systemic inflammation response was investigated in pre‐LPS treated (Day 1) and LPS treated (Day 5) fetal PBMC transcriptomes. The data have been deposited in the National Center for Biotechnology Information's Gene Expression Omnibus (accession # GSE284345). PBMC transcriptomes from both days were evenly distributed across three principal components (PCs; Figure 3b). A similar variation found in transcriptomic data between the two sampling days highlights their separation, with PC1 contributing 18.69%, PC2 contributing 17.4%, and PC3 contributing 12.24%. Eighty DEGs (FDR *q* < 0.05 and fold change >1.5) are represented in the volcano plot (Figure 3c). Fifty‐nine genes were upregulated, and 21 genes were downregulated in LPS‐exposed PBMCs (Day 5) compared to pre‐LPS exposed (Day 1) (Table S1). In response to LPS, 40 DEGs were aligned with inflammation signature pathways (Figure 3d). Expression levels for pre‐LPS (Day 1) and LPS (Day 5) inflammatory genes are presented in a heat map (Figure 3e), which indicates that most of the LPS responsive DEGs are associated with the inflammatory gene signature.

**FIGURE 4:**
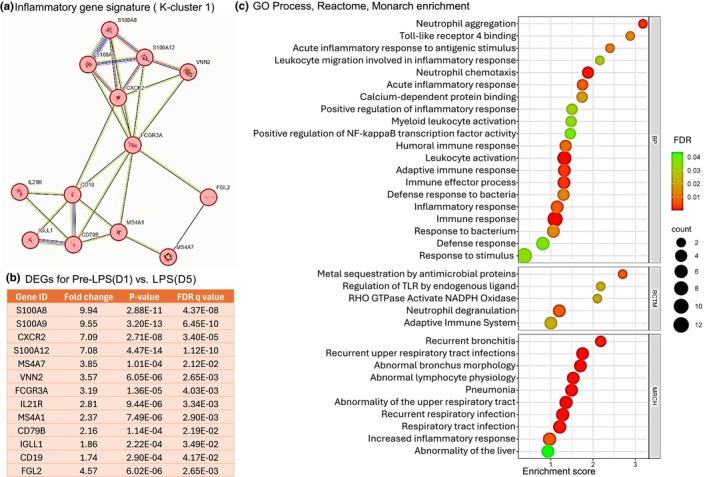
K‐Cluster 1 from 40 DEGs associated with inflammatory gene signatures. LPS stimulation upregulated several prominent genes in PBMCs associated with chronic inflammation. (a) K‐cluster 1 represents a node of 13 DEGs in the String Analysis. (b) Table of these 13 genes with gene identification, fold change (log_2_), *p*‐values, and FDR *q* value. These data demonstrate a robust upregulation of 13 genes in K‐cluster 1. (c) String protein–protein interaction analysis reveals pathway enrichment from GO process, Reactome, and Monarch. Each pathway is represented on the *y*‐axis with its associated gene numbers, while the *x*‐axis displays the enrichment score for each pathway.

**FIGURE 5:**
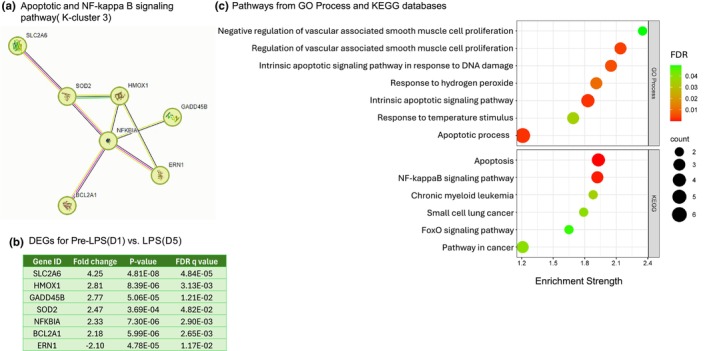
K‐Cluster 3 from 40 DEGs associated with inflammatory gene signatures is associated with apoptotic and NF‐Kappa B signaling. (a) K‐cluster 3 represents a node of 7 DEGs. (b) Table of these 7 DEGs with gene identification, fold change, *p*‐value, and FDR q value. Six DEGs were upregulated, and one was downregulated in K‐cluster 3. (c) String protein–protein interaction database reveals pathway enrichment analysis for GO process and KEGG database. Pathways are listed on the *y*‐axis with their associated gene numbers, and the *x*‐axis displays the enrichment score.

**FIGURE 6:**
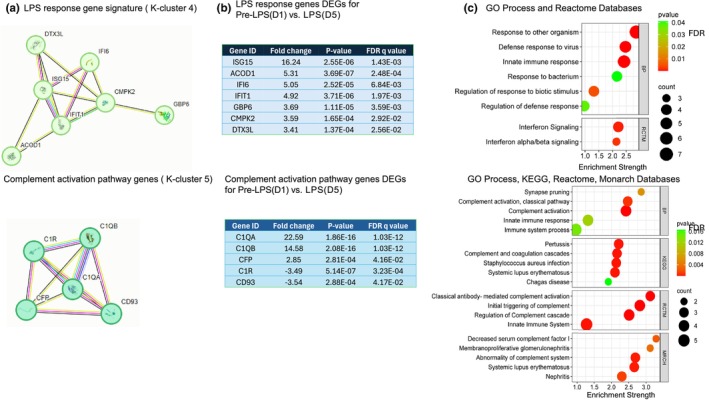
K‐Cluster 4 and K‐Cluster 5 from 40 DEGs associated with inflammatory gene signatures are associated with LPS response gene signature and Complement activation genes, respectively. (a) K‐cluster 4 represents a node of 7 genes from LPS response gene signature; K‐cluster 5 represents a node of 5 genes from Complement activation gene signature; (b) Tables for the 7 DEGs in LPS response genes and 5 DEGs for Complement Activation genes are presented with gene identifications, fold change, *p*‐value, and FDR *q* value. (c) String protein–protein interaction enrichment analysis for GO process, Reactome, KEGG, or Monarch databases is presented. Pathways are listed on the *y*‐axis with their associated gene numbers, and the *x*‐axis displays the enrichment score.

Association to inflammation and immune response is supported by gene enrichment analysis using the protein–protein interaction (PPI) network and further K‐clustering analysis of the 40 DEGs associated with the inflammation gene signature pathway. Our analysis of the PPI network led to the identification of 38 nodes, average local clustering coefficient 0.673, PPI enrichment (*p* < 1.11e‐16). K‐clustering analysis segmented the PPI network into five interactive modules (Tables S2–S7). Notably, several DEGs and the key genes within each module have been previously reported to be involved in the characteristic response to inflammation and immune functions. A detailed analysis of K cluster 1 identified 13 genes primarily associated with the inflammatory response (Figure 4a). These 13 DEGs in cluster 1 were upregulated (Figure 4b; *p* < 0.05, FDR *q* < 0.05) and shared enrichment with several immune‐related pathways (FDR *q* < 0.05), which are illustrated by their gene number and enrichment score (Figure 4c). A similar comprehensive analysis was performed on K‐cluster 3, which comprises 7 genes, as illustrated in Figure 5a–c, along with K‐cluster 4 and K‐cluster 5, depicted in Figure 6a–c. The DEGs in K‐cluster 3 reveal several genes related to apoptosis and NF‐kappa signaling. K‐cluster 4 and K‐cluster 5 showed these nodes of DEGs were associated with the immune response to lipopolysaccharide (LPS) stimulation and complement activation.

### 3.3 No differences in fetal weights

Fetal age at necropsy was similar between the LPS‐exposed (127 ± 2 dGA, 2 males and 3 females) and LPS‐naïve fetuses (129 ± 1 dGA, 2 males and 3 females). Fetal weight was not different between LPS‐exposed (2.8 ± 0.4 kg) and LPS‐naïve fetuses (3.0 ± 0.5 kg).

### 3.4 LPS challenges stimulate proinflammatory cytokine expression

**FIGURE 7:**
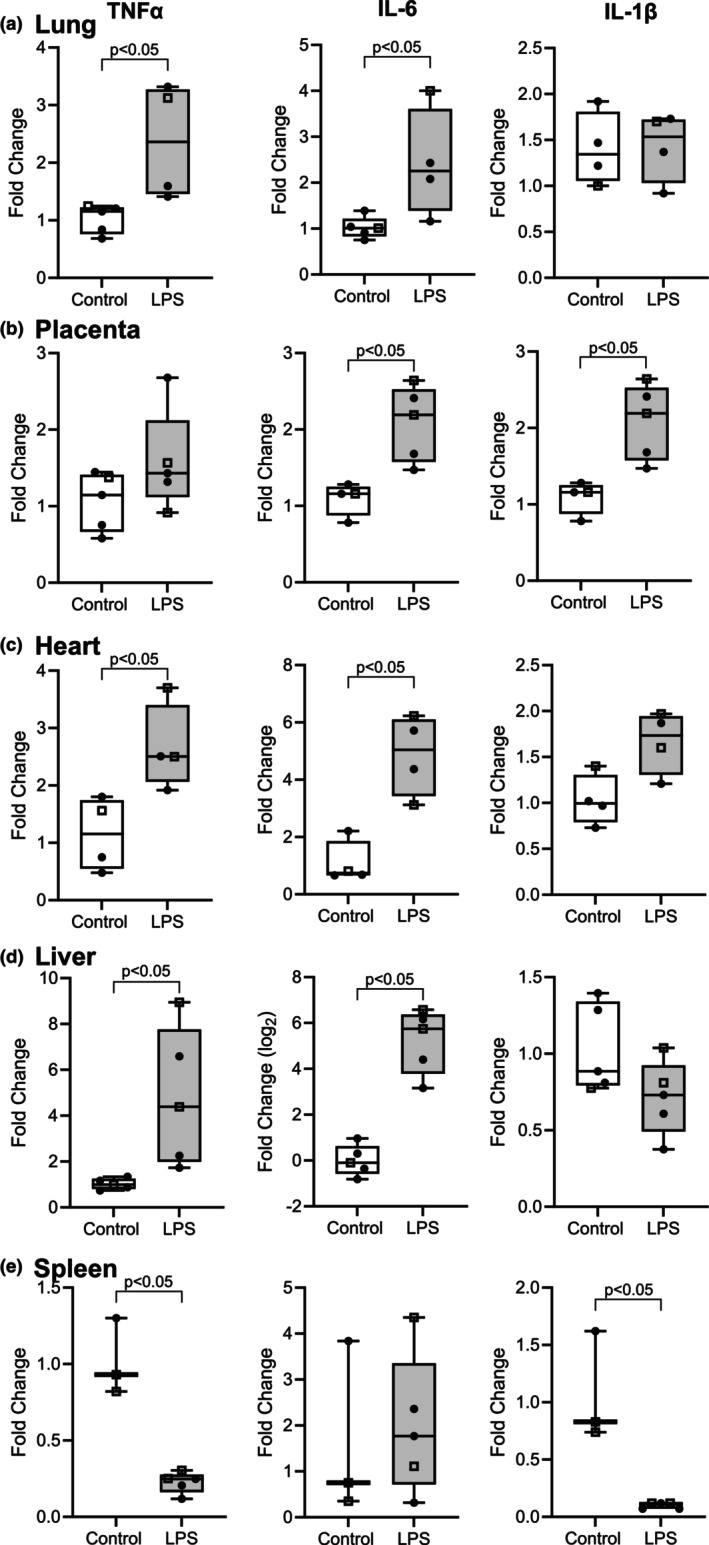
Elevated pro‐inflammatory cytokine expression in fetal organs after repeated LPS challenges. Naïve control fetal tissues were compared to LPS‐treated fetal sheep tissues (*n* ≥ 3). Quantitative PCR for TNFα, IL‐6, and IL‐β mRNA concentrations were measured in lung (a), placenta (b), heart (c), liver (d), and spleen (e). Gene of interest expression was normalized using the geometric mean of ovine reference genes and presented as the fold change relative to the naïve controls ($2^{-\Delta \Delta C_{t}}$). Statistical differences were determined with a two‐tailed T‐test on the ΔC(t) values and are indicated. Fetal sex is designated by symbols (open square for male; fill circle for female).

Pro‐inflammatory cytokine mRNA expression levels for TNFα, IL‐6, and IL‐1β were measured in fetal lung, heart, placenta, liver, and spleen (Figure 7). In lung, heart, and liver, the concentration of TNFα transcripts was elevated compared to tissues from naïve controls. TNFα concentrations decreased in the spleen and were not different in the placenta. IL‐6 mRNA concentrations were greater in heart, placenta, and liver of the LPS fetus compared to the control fetus. IL‐6 increased in the lung and was unchanged in the spleen. IL‐1β mRNA concentrations were greater in the placenta of the LPS fetus versus control. IL‐1β mRNA concentrations were lower in the spleen of the LPS fetus and unaffected in the lung, heart, and liver.

## 4 DISCUSSION

In this study, we established a new model of FIRS in fetal sheep that consistently and effectively elicits a robust physiological response to increasing sublethal LPS doses. Our findings support the hypothesis that repeated increasing doses of LPS, when given intravenously to fetal sheep, overcome their tachyphylaxis to LPS. Notably, increasing subsequent LPS doses produced robust systemic inflammatory responses, where the magnitude of physiological responses was sustained across all four LPS challenges that increased 50‐fold. The PBMC results also show that the advancing days and higher doses of LPS stimulate FIRS because PBMC concentrations increased, and the transcriptomic analysis indicates a strong inflammatory response to LPS exposure. To further characterize the FIRS response, expression levels of pro‐inflammatory cytokines were upregulated in the lung, heart, placenta, and liver, but, as expected, downregulated in the spleen of LPS‐challenged fetuses compared to naïve fetal sheep. Thus, we have established a new model of chorioamnionitis‐induced FIRS that replicates a clinical setting with prolonged exposure to infection.

An initial low dose LPS bolus (0.3 μg/kg) induced a physiological response and established endotoxin tolerance that allowed fetuses to survive subsequent doses of 5, 10, and 50 times the initial dose. Repeated higher doses of LPS led to an acute physiological response where pH and pO_2_ levels were decreased, while pCO_2_, lactate, and cortisol concentrations increased at 3 and 5 h after each bolus. These physiological alterations such as acidosis, hypoxia, hyperlactatemia, and endocrine stress are hallmarks of inflammation that illustrate fetal stress (Mathai et al., 2013; Turkoglu et al., 2023; Zarate et al., 2020). Strikingly, the magnitude of physiological responses to the increasing doses was preserved across all four LPS challenges because there was no interaction between hour and day, which demonstrates that the responses for all measurements were similar. However, the daily lactate average was lower on Day 5 compared to Day 3 and indicates that the response in lactate was lower even with the 10‐fold increase in LPS (Figure 2n). The diminished LPS induction of hyperlactatemia on Day 5 is partially explained by the greater cortisol concentrations (Figure 2o) or by greater hypoxemia, which both promote lactate clearance (Figure 2M) (Hacker et al., 2023; Wilkening et al., 1993). Elevated cortisol can facilitate lactate clearance through mechanisms like increased gluconeogenesis (Timmerman et al., 2000). Furthermore, cortisol can promote anti‐inflammatory effects and result in metabolic shifts to lower lactate concentrations (Silva et al., 2019).

LPS stimulates transcription of pro‐inflammatory cytokines and secretion by activating toll‐like receptor 4 (TLR4) present on granulocytes, monocytes, and lymphocytes in fetal blood (Aksel & Akyuz, 2021; Dorner et al., 2009). Thus, PBMC concentrations, along with transcriptional analysis, were used to evaluate the systemic FIRS response during repeated LPS challenges. PBMC concentrations increased 48 h after the first bolus (data not shown) and remained elevated but stable after the second and third boluses collected on the 4th and 5th days (Figure 3a). These data demonstrate that our model effectively triggers an inflammatory response in PBMCs that reaches near maximal levels by the 4th day with two LPS boluses on days 1 and 3. Stabilization of PBMC numbers between the 4th and 5th day might be due to endotoxin tolerance, the delayed nature of the inflammatory response, and the body's negative feedback mechanisms that help stabilize the response over time (Biswas & Lopez‐Collazo, 2009). Importantly, the lack of physiological responses in ewe was consistent with our hypothesis that the maternal immune system will not influence the fetal response as maternal circulation is isolated from any fetal intravenous LPS exposure.

Transcriptomic analysis on PBMCs comparing cells isolated pre‐LPS (Day 1) and after LPS‐exposure (Day 5) showed that intravenous LPS administration, in addition to increasing PBMC concentrations, also upregulated the expression of genes specifically associated with inflammatory gene signatures, apoptosis, NFκB, LPS response genes, and the complement activation pathway. Differentially expressed genes such as CXCR2, S100A8, S100A12, IFI6, NFKBIA, BCL2A1, IL21R, and HMOX1 were shown to be upregulated and are all regulators of immune responses and inflammation. NFκB signaling is also associated with the activation of inflammatory cytokines like TNFα, IL‐1β, and IL‐6 (Liu et al., 2017). Enriched processes or pathways for the k‐clusters presented identify innate immunity or TLR regulation, which supports endotoxin‐induced inflammatory response in fetal PBMCs (Ahmed et al., 2023; Galanos & Freudenberg, 1993; Ngkelo et al., 2012; Wright & Kirpalani, 2011). Most of the DEGs found in our data are actively expressed in humans, indicating similar molecular functions between our chorioamnionitis model in sheep induced with LPS and in human fetuses with chorioamnionitis (Weitkamp et al., 2016). Specifically, 14 DEGs from fetal sheep PBMCs were orthologs and closely resemble those expressed in human infants born with chorioamnionitis. Together, the transcriptomic analysis of the PBMCs indicates a strong inflammatory response to higher doses of LPS in sheep fetuses.

Elevated cytokine mRNA expression in selected tissues (lung, liver, heart, and placenta) further indicates systemic inflammation in LPS‐exposed fetuses. FIRS is associated with respiratory distress syndrome and bronchopulmonary dysplasia in human neonates and has been extensively studied in intraamniotic sheep models (Ericson & Laughon, 2015; Muraskas et al., 2020; Zarate et al., 2020). In lung tissue, TNFα expression was increased and paralleled by moderately higher IL‐6 expression. Moreover, this chorioamnionitis model is associated with hepatic inflammation, which is also demonstrated in this study with higher TNFα and IL‐6 concentrations (Gomez‐Lopez et al., 2019; Gotsch et al., 2007; Kallapur et al., 2014; Romero et al., 1989). Fetal liver influences the innate inflammatory response and houses a unique macrophage called Kupffer cells (Wen et al., 2021; Zarate et al., 2020). Inflammation of the heart and placenta was also demonstrated with greater IL‐6 expression. Although we recognized the limitation of measuring RNA versus protein, these findings indicate systemic inflammation in several fetal tissues after the four LPS challenges.

In human fetus, neutrophils increase in the spleen during chorioamnionitis, while also displaying a decreased percentage of T and B cells (Stallmach & Karolyi, 1994). Additionally, severe shrinkage of the thymus and spleen has been observed, contributing to the depletion of leukocytes induced by the onset of sepsis (Toti et al., 2004). Previous studies in sheep and rhesus Macaques with intra‐amniotic LPS administration showed that the total number of macrophages and T‐regulatory cells decreased in lymphoid tissues like the spleen and gut (Kallapur et al., 2013; Kuypers et al., 2012; Lee et al., 2011; Rueda et al., 2016). Similarly, our LPS model in fetal sheep led to a decrease in cytokine gene expression in the spleen and aligns with those previous findings. This might be attributed to thymus modulation and diminished expression of FOXP3+ cells in LPS‐exposed lambs, which can produce pro‐inflammatory cytokines like IL‐17 (Kallapur et al., 2013; Kunzmann et al., 2010; Rueda et al., 2016). Another explanation could be the migration of immune effector cells to the site of inflammation (Lee et al., 2011).

Our experimental approach with repeated fetal IV boluses of LPS offers several new advantages for evaluating immunomodulating therapeutics in fetuses with FIRS. Specifically, we demonstrate a robust physiological response with each increasing LPS dose, a high survival rate of fetuses over a five‐day period, and a comprehensive assessment of multiple organ establishment of FIRS. Activation of inflammatory cascades in the fetus is a crucial factor that drives the adverse outcomes in fetuses affected with chorioamnionitis (Ahmed et al., 2023; Gisslen et al., 2014; Heymans et al., 2021). Thus, systemic inflammation was verified with elevated pro‐inflammatory cytokines in multiple tissues, along with increases in PBMC concentrations and DEGs enrichment of inflammatory gene signatures. In conclusion, this model with a high survival rate offers significant promise for enhancing the understanding of systemic inflammation and the development of therapeutic strategies to mitigate adverse effects caused by FIRS.

## AUTHOR CONTRIBUTIONS

AM, SK, AG, and SWL conceived and designed experiments; SK, AG, ART, RILR, SKT, NZ, AM, and SWL performed experiments, analyzed data, interpreted results of experiments, and prepared figures; SK drafted the manuscript; all authors edited and revised the manuscript; all authors approved the final version of the manuscript.

## FUNDING INFORMATION

This work was supported by Flinn Foundation #23–12,143.

## DISCLAIMERS

The authors have no competing interests.

## ETHICS STATEMENT

Animal protocols were approved by the Institutional Animal Care and Use Committee at the University of Arizona. All experiment followed the guidelines set by the US National Research Council's “Guide for The Care and Use of Laboratory Animals” and the US Public Health Service's “Policy on Humane Care and Use of Laboratory Animals.”

## Supporting information


Table S1.

## Data Availability

The data that support the findings of this study are openly available in the National Center for Biotechnology Information's Gene Expression Omnibus (accession # GSE284345).
